# Retinoic Acid-Related Orphan Receptors (RORs): Regulatory Functions in Immunity, Development, Circadian Rhythm, and Metabolism

**DOI:** 10.11131/2015/101185

**Published:** 2015-12-16

**Authors:** Donald N. Cook, Hong Soon Kang, Anton M. Jetten

**Affiliations:** 1 Immunogenetics Section, Immunity, Inflammation and Disease Laboratory, National Institute of Environmental Health Sciences, National Institutes of Health, Research Triangle Park, NC 27709, USA; 2 Cell Biology Section, Immunity, Inflammation and Disease Laboratory, National Institute of Environmental Health Sciences, National Institutes of Health, Research Triangle Park, NC 27709, USA

**Keywords:** retinoic acid-related orphan receptor, ROR*γ*, ROR*α*, ROR*β*, immunity, Th17 cells, innate lymphoid cells, autoimmune disease, retina, brain, cancer, metabolism, glucose homeostasis, lipid metabolism, insulin sensitivity, diabetes, circadian clock, agonists, cholesterol biosynthesis, autism, agonists, antagonist, transcription

## Abstract

In this overview, we provide an update on recent progress made in understanding the mechanisms of action, physiological functions, and roles in disease of retinoic acid related orphan receptors (RORs). We are particularly focusing on their roles in the regulation of adaptive and innate immunity, brain function, retinal development, cancer, glucose and lipid metabolism, circadian rhythm, metabolic and inflammatory diseases and neuropsychiatric disorders. We also summarize the current status of ROR agonists and inverse agonists, including their regulation of ROR activity and their therapeutic potential for management of various diseases in which RORs have been implicated.

## 1. Introduction

The retinoic acid-related orphan receptors alpha, beta, and gamma (ROR*α-γ*, RORA-C or NR1F1–3) constitute a subfamily of nuclear receptors that function as ligand-dependent transcription factors [1–3]. By using different promoters and/or alternative splicing, each ROR gene produces several isoforms that vary only at their N-terminus. The *RORa* gene generates four isoforms, ROR*α*1–4, while *RORb* and *RORc* each generate two isoforms [4–10]. Most isoforms exhibit a distinct tissue-specific pattern of expression and regulate different biological processes and target genes. For example, the expression of ROR*γ*2, commonly referred to as ROR*γ*t, is restricted to several immune cell types, while ROR*γ*1 is only expressed in various peripheral tissues, including liver, adipose tissue, skeletal muscle, and kidney [1, 7, 8, 11–14]. RORs are critical in the regulation of many physiological processes, including immunity, circadian rhythm, embryonic development, and several metabolic pathways, and have been implicated in several pathologies associated with those processes.

RORs exhibit a domain structure that is typical of nuclear receptors and contain an N-terminal domain, a highly conserved DNA-binding domain (DBD) consisting of two C2-C2 zinc finger motifs, a ligand-binding domain (LBD), and a hinge domain spacing the DBD and LBD [1]. The RORs regulate gene transcription by binding as monomers to ROR response elements (ROREs) consisting of the RGGTCA consensus preceded by an A/T-rich sequence in the regulatory regions of target genes [6, 15]. The ability to bind ROREs is shared with several other nuclear receptors, including the transcriptional repressors Rev-Erb*α* and Rev-Erb*β* (NR1D1–2) [16]. By competing for RORE binding, these receptors can antagonize each other’s effects on transcription. For example, crosstalk between RORs and Rev-Erbs plays a role in the transcriptional regulation of a number of metabolic and clock genes [9, 16– 25].

Relatively little is known about posttranslational modifications and upstream signaling pathways that modulate ROR transcription activity. Protein kinase A (PKA) has been reported to activate ROR*α*4, and although PKA phosphorylates ROR*α*4 at Ser99, mutation of this site had little influence on ROR*α*4 transcriptional activity [26], while phosphorylation of ROR*α*4 at Thr128 by ERK2 enhances its transcriptional activity [27]. PGE2/PKC*α*-dependent phosphorylation of ROR*α* has been reported to attenuate Wnt target gene expression in colon cancer cells [28], while sumoylation of ROR*α* enhanced its transcriptional activity [29]. A recent study demonstrated that the deubiquitinase, DUB, interacts with and stabilizes the ubiquitin ligase UBR5 in response to TGF-*β* signaling [30]. This results in an increase in ROR*γ*t ubiquitination that leads to reduced ROR*γ*t stability and diminished transactivation of ROR*γ*t target genes in T-helper type 17 (Th17) cells. Another study reported that the protein deacetylase, Sirtuin 1 (SIRT1), deacetylates ROR*γ*t and increases its transcriptional activity, thereby enhancing Th17 generation [31].

Reports showing that cholesterol and cholesterol sulfate, as well as a series of other small molecules, were able to bind the LBD of RORs and modulate its transcriptional activity indicated that RORs function as ligand-dependent transcription factors [2, 25, 32, 33]. Recently, several intermediates of the cholesterol biosynthetic pathway were reported to act as endogenous agonists of ROR*γ* [34, 35]. These studies revealed that ROR*γ* transcriptional activity and the physiological processes it regulates, can be controlled by changes in the intracellular pool of these sterol intermediates. In addition, these discoveries raised the possibility that ROR ligands might be valuable in the development of new therapeutic strategies for diseases in which RORs are implicated, including various inflammatory and metabolic diseases and neuropsychiatric disorders. In this review, we summarize several areas of ROR research in which recently significant progress has been made.

## 2. RORs in Adaptive Immunity

**Figure 1:**
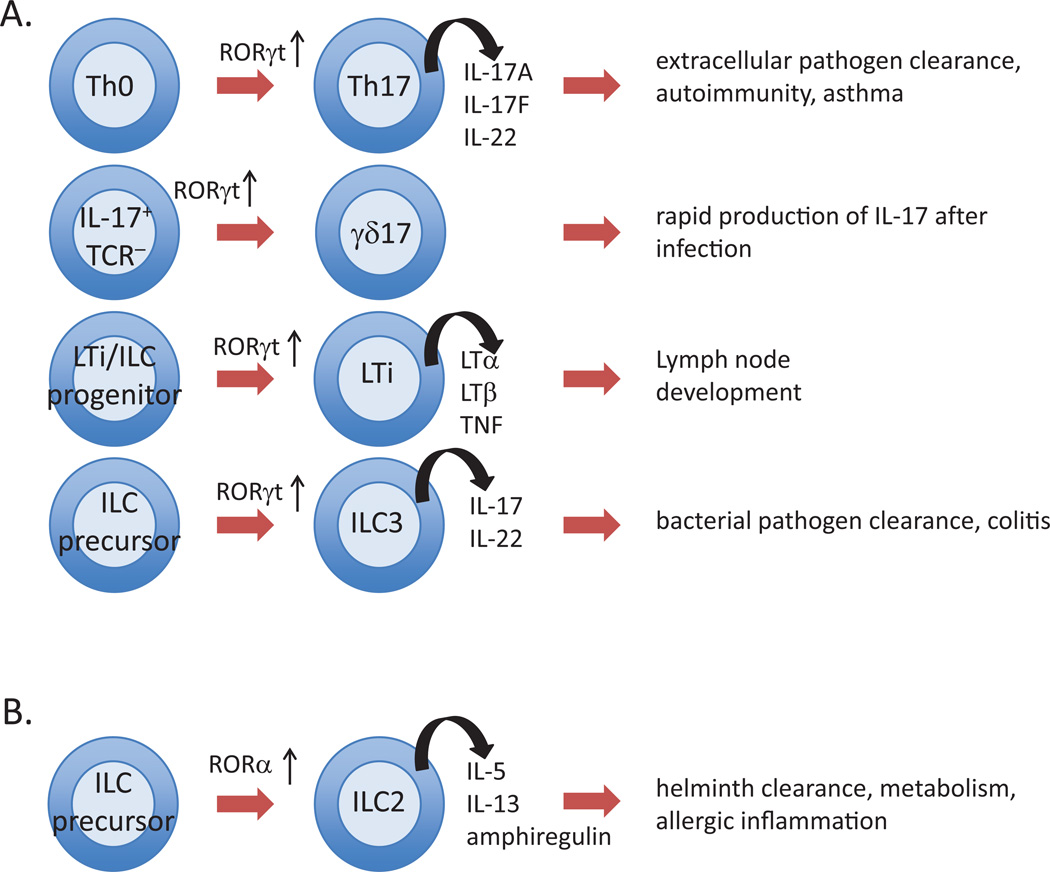
Multiple functions of RORs in lymphocyte development A. Roles of ROR*γ*t in the development of Th17 cells, *γδ*-17 T cells, lymphoid tissue inducer (LTi) cells and innate lymphoid cells 3 (ILC3) cells. (B) Role of ROR*α* in the development of ILC2 cells. ROR*α* has also a role in the regulation of Th17 cells.

The innate and adaptive immune systems are highly integrated and serve to protect the host from being overwhelmed by pathogen invasion. Innate immune responses are immediate and utilize germline-encoded receptors to recognize and respond to pathogens, whereas adaptive immunity is a delayed response that requires expansion of a small number of cells bearing antigen-specific receptors on the surface of lymphocytes. Genetically modified mice lacking ROR*α* or ROR*γ*t have revealed that each receptor plays a key role in the development of several immune cells and are critical for some immune responses (Figure 1).

Lymphoid progenitor cells undergo several stages of differentiation in the thymus prior to becoming mature T cells. These stages can be identified in part by display of CD4 and CD8 on the cell surface. ROR*γ*t is selectively expressed in T cells that display both CD4 and CD8, typically called double positive (DP) cells. ROR*γ*t is required in these cells for expression of the anti-apoptotic gene *Bcl-X_L_* [36–40]. *Bcl-X_L_* expression is repressed in DP thymocytes of ROR*γ* null mice, resulting in accelerated apoptosis *in vivo* and *in vitro*. Consequently, thymi of ROR*γ* null mice have reduced numbers of DP cells and their descendants, including single positive (SP) mature CD4^+^ T helper cells (Th) and CD8^+^ cytotoxic cells.

**Figure 2:**
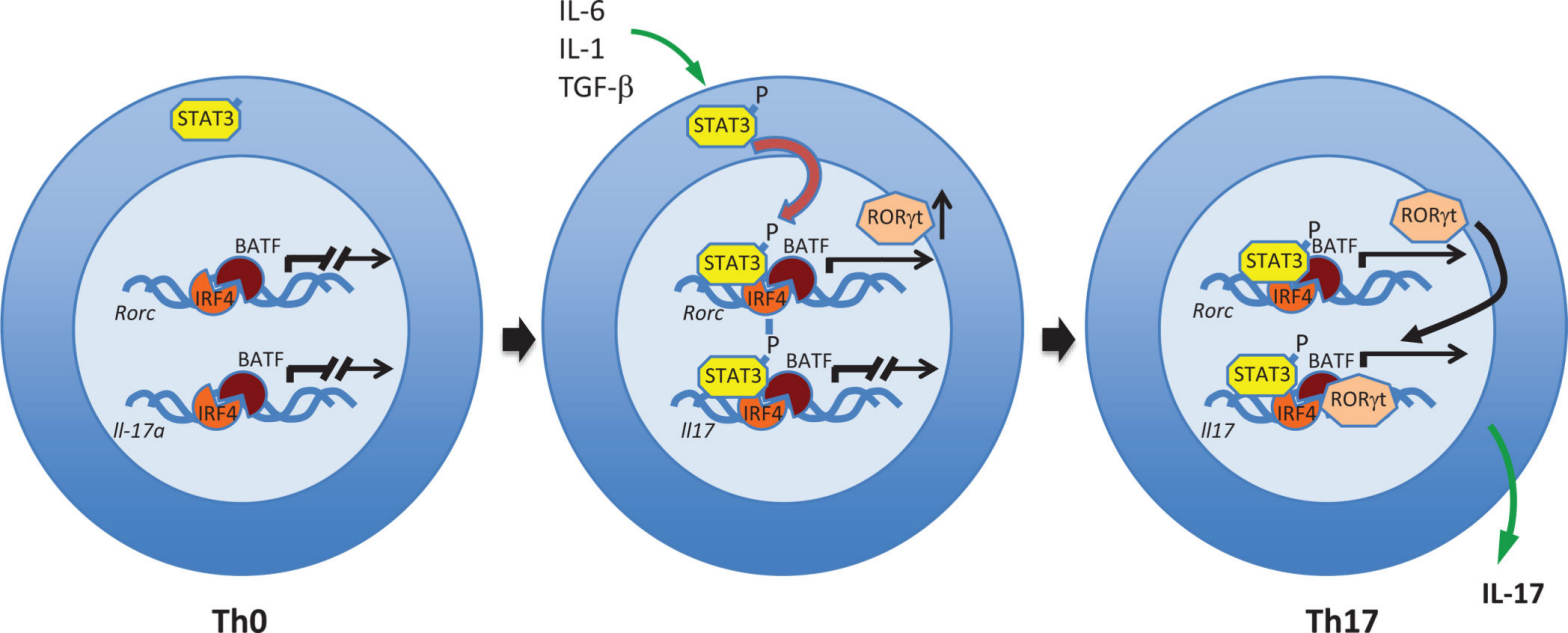
ROR*γ*t-dependent induction of Th17 differentiation and Th17-associated genes In Th0 cells, IRF4 and BATF are bound to chromatin near Th17-associated genes, but the loci are transcriptionally silent. Upon exposure to cytokines, such as IL-6, STAT-3 becomes phosphorylated and transfers to the nucleus, where it binds DNA near IRF4 and BATF and induces *Rorc* transcription. ROR*γ*t can then join the IRF4/BATF/STAT3 transcription factor complex and induce expression of Th17-associated genes, such as *Il17* and *Il23r*.

Mature, but naïve CD4^+^T (Th0) cells can be differentially polarized to produce the cytokines characteristic of Th1, Th2 and Th17 cells [1, 41]. ROR*γ*t is required for the development of Th17 cells [12, 13, 42–45], whose name reflects their ability to produce the cytokines IL-17A and IL-17F, as well as IL-21 and IL-22. Like ROR*γ*t, ROR*α* can also contribute to Th17 development and acts synergistically with ROR*γ*t in this regard [13, 44]. Th17 cells protect against extracellular pathogens, but are also associated with various diseases, such as rheumatoid arthritis, inflammatory bowel disease, multiple sclerosis and asthma [46, 47]. Forced expression of ROR*γ*t is sufficient for the induction of several Th17-associated genes, including *Il17, Il22, Ccr6*, and the IL-23 receptor (*Il23r*) [12, 48, 49]. Combinations of promoter and chromatin immunoprecipitation (ChIP) analysis, and cistrome mapping showed that ROR*γ*t is recruited to ROREs in several Th17 marker genes, including *Il17a, Il17f, Irf4* and *Il23r*, and directly regulate their transcription [43, 44, 50]. However, in addition to ROR*γ*t, several other transcriptional factors are necessary to induce the full Th17 differentiation program, including BATF [51], IRF4 [52], and STAT3 [53]. Recent advances in ChIP and RNA-seq technologies have shed light on the hierarchy and order of transcription factor occupancy during Th17 differentiation [54, 55]. In Th0 cells, the transcription factors, IRF4 and BATF, are cooperatively bound to overlapping sites in chromatin near several genes, including *Rorc* and *Il17*, thereby increasing chromatin accessibility to other transcription factors (Figure 2). In response to TGF*β* and IL-6, STAT3 becomes phosphorylated (pSTAT3) and moves to the nucleus, where it binds to chromatin and induces expression of *Rorc*. ROR*γ*t and pSTAT3 then cooperate with IRF4 or BATF and other factors to increase expression of Th17-associated genes, including *Il17a, Il17f, Il23r, Ccl20* and *Il1r1* [55]. Thus, IRF4 and BATF have broad and self-reinforcing effects on chromatin remodeling, whereas ROR*γ*t specifically regulates a relatively small number of key Th17-associated genes in a manner potentially responsive to environmental cues. In addition to transcriptional control by IRF4/BATF/STAT4, the PI3K-Akt-mTORC1-S6K1/1 cell signaling axis has also been linked to the control of Th17 differentiation by ROR*γ*t [56]. Activation of PI3K-Akt-mTORC1 induces ribosomal protein S6 kinase *β*2 (*RPS6KB2*) expression that subsequently promotes the nuclear localization of ROR*γ*t and ROR*γ*t-mediated Th17 differentiation.

The differentiation of Th0 cells into Treg and Th17 cells is dependent on the balance between the level of expression of Foxp3 and ROR*γ*t, respectively. Through its interaction with ROR*γ*t, Foxp3 inhibits ROR*γ*t function and promotes Treg differentiation [1, 57, 58]. This balance is controlled by the concentration of specific cytokines in the environmental milieu. *Foxp3* expression and consequent Treg development is favored in cultures containing high levels of TGF-*β*, IL-2 and retinoic acid, whereas Th17 development is promoted by low amounts of TGF-*β* in combination with the proinflammatory cytokines, IL-6 and IL-1 [58– 60]. IL-1 can repress the suppressor of cytokine signaling 3 (SOCS3), an inhibitor of STAT3 phosphorylation [61], thereby increasing *Rorc* expression. Th17 cells share an overlapping developmental program with that of inducible regulatory T cells (iTregs) [62]. In the small intestine, a number of ROR*γ*t^+^Foxp3^+^ T cells do not produce IL-17, but instead express IL-10. These ROR*γ*t^+^ Tregs develop outside the thymus and require gut microbiota for their development. In addition, dietary vitamin A favors the generation of these ROR*γ*t^+^ Tregs over that of Th17 cells [62]. ROR*γ*t^+^ Treg cells regulate Th2 cells - but not Th1 or Th17 cells -through a CTLA4-dependent regulation of CD80 and CD86 on dendritic cells.

Th17 and IL17 have been implicated in several inflammatory and autoimmune diseases. Mice lacking ROR*γ* are partially protected against the development of diseases, including autoimmune diseases such as experimental autoimmune encephalomyelitis (EAE) and type II collagen-induced arthritis, as well as allergen-induced lung inflammation [12, 44, 58, 63]. Mice lacking both ROR*α* and ROR*γ* are greatly protected from EAE [44]. Although IL-17A, IL-17F and IL-22 are the signature cytokines of Th17 cells, they appear not to be sufficient for pathogenicity in EAE [64, 65]. In this model, ROR*γ*t-dependent production of granulocyte macrophage colony stimulating factor (GM-CSF) is reported to drive the effector phase of neuroinflammation [66, 67]. However, the molecular requirements of pathogenicity might depend on the disease model because either IL-17A or IL-17F is required for Th17 cell-mediated intestinal inflammation [68]. Together, these studies raise the possibility that ROR*γ* antagonists might be useful in the management of autoimmune disease.

## 3. RORs in Innate Immunity

Like conventional *αβ* T cell receptor (TCR)^+^ cells, T cells expressing the *γ* and *δ* TCR chains (*γδ* T cells) develop in the thymus, but they have a more limited repertoire than *αβ* TCR^+^ cells and lack major histocompatibility complex (MHC) restriction [69]. Many *γδ* T cells express IL-17 and are thus termed *γδ*-17 T cells [70, 71]. Unlike *αβ* TCR Th17 cells, which acquire effector functions only after encountering their cognate antigens in peripheral tissue, many *γδ*-17 cells express IL-17 very early during their development in the thymus, even prior to TCR rearrangement [70, 71]. Thus, *γδ*-17 cells have the potential to be a major cell source of IL-17, especially during the early phases of disease, prior to the development of antigen-specific Th17 cells [14, 72]. There are some commonalities, but also differences in the molecular pathways leading to *γδ*-17 and Th17 cells. For both cell types, ROR*γ*t is critically important. Thus, mice deficient for ROR*γ*t lack *γδ*-17 cells in peripheral organs and lymphoid tissues [12]. However, the induction of ROR*γ*t in Th17 cells requires the canonical c-Rel-dependent NF-kB pathway, whereas *γδ*-17 cells require RelB and the noncanonical NF-kB pathway [73]. In addition, IRF4 is required for the induction of Th17 cells, but this transcription factor is dispensable for the development of *γδ*-17 T cells.

ROR*α* and ROR*γ* also play a critical role in the generation of innate lymphoid cells (ILCs). ILCs are a heterogeneous population of cells that possess the typical lymphoid cell morphology, but lack some cell surface molecules typically seen on lymphocytes [74]. In particular, ILCs lack TCRs and the associated CD3 complex found on conventional T cells. Consequently, ILCs cannot recognize specific antigens, and instead respond to cytokines produced during innate immune responses. ILCs have been classified into three groups, based on their cytokine production profiles and the transcription factors that regulate their development [75]. The cytokines produced by each of these groups mirrors those produced by specific T helper (Th) cell types: Group 1 ILCs and Th1 cells produce IFN-*γ*, Group 2 ILCs and Th2 cells produce IL-5 and IL-13, and Group 3 ILCs and Th17 cells produce IL-17A, IL17F and IL-22.

ROR*γ*t is required for the development of all ILC3s, a heterogeneous population of cells that also depends on IL-7 for their development. The first discovered member of the Group 3 ILCs is the lymphoid tissue inducer (LTi) cell, a type of CD4^+^CD3^−^ cell that displays transmembrane lymphotoxin *α*1*β*2 [76, 77]. These cells are required for the development of secondary lymphoid organs, Peyer’s patches, and intestinal lymphoid follicles [78–81]. ROR*γ*t-deficient mice lack these cells and therefore do not develop secondary lymphoid organs [1, 36, 37, 82]. Recently, retinoic acid (RA) was found to control LTi cell maturation upstream of ROR*γ*t by positively regulating ROR*γ*t expression directly through the recruitment of RA receptors (RARs) to the promoter region of ROR*γ*t [83]. Impairment of LTi maturation in cells defective in RA signaling can be rescued by the exogenous expression of ROR*γ*t. More recently, other ILC3 subpopulations have been identified that, like Th17 cells, are abundant in the gut and can produce IL-17A, IL-17F, IL-22, GM-CSF, and TNF, suggesting their importance for clearing extra-cellular pathogens. Gata-3 is critical for the development of gut ROR*γ*t^+^ ILC3s subsets [84]. At least some ILC3s can transition to ILC1 cells [85], reminiscent of Th17 cell conversion to a Th1-like phenotype. This transition, which is accompanied by elevated levels of TBX21, is driven by cytokine signals in the cellular milieu.

Group 2 ILCs are the most homogeneous group of the ILCs and are dependent on ROR*α*, but not ROR*γ*t, for their development [86, 87]. They display the cell surface markers IL-7Ra (CD127), IL-2RA (CD25), Sca-1, KLRG1 and the IL-33 receptor, ST2. ILC2s are defined based on their ability to produce an array of type 2 cytokines, including IL-4, IL-5, IL-9, and IL-13, as well the cytokine, amphiregulin, and are implicated in helminth clearance and allergic inflammation [88]. Several single nucleotide polymorphisms (SNPs) within the *RORA* gene are associated with increased susceptibility to asthma [89–91], and ROR*α* null mice and ILC2-deficient mice generated by ROR*α*-deficient bone marrow transplants have reduced type 2 cytokine production and partial protection from airway hyper-reactivity [87, 92]. ROR*α* expression was significantly upregulated in patients with therapy-resistant asthma [93]. ROR*α* expression was also found to be significantly elevated in skin from patients with atopic dermatitis (AD), while in an experimental model of AD-like inflammation ROR*α*-deficient mice exhibit a profound deficit in ILC2 cells and significantly reduced allergic skin inflammation [88]. Together, these observations indicate that ROR*α* plays a critical role in ILC2 cell lineage determination and control of allergy-induced inflammation in multiple tissues.

ROR*α* also contributes to immune function in the intestinal epithelium by controlling the diurnal regulation of several pathogen recognition receptors, including Nod2 and various Toll-like receptors [94]. The expression of these genes in the intestine is reduced in ROR*α*-deficient mice, particularly when ROR*α* expression is at its highest and bound to promoter regions of these genes. Other genes whose diurnal expression is directly controlled by ROR*α* include interleukin-1 receptor-associated kinase 1 (IRAK1), Toll-interleukin 1 receptor domain containing adaptor protein (TIRAP), and the clock genes, Bmal1 and nuclear factor, interleukin 3 regulated (NFIL3 or E4BP4). The decreased expression of ROR*α* in the intestine of microbiota-depleted mice further provides additional evidence of a relationship between ROR*α*, commensal bacteria and diurnal regulation of immune-related genes in the gut [94].

## 4. ROR Functions in Brain and Retina

ROR*α* is highly expressed and developmentally regulated in several regions of the brain, including cerebellar Purkinje cells and the thalamus [1, 95–97]. Genetically modified mice, in which *RORα* is disrupted, and ROR*α*-deficient *staggerer* (*RORα^sg/sg^*) mice display severe cerebellar ataxia due to cerebellar neurodegeneration [98–100]. Further characterization of these mice revealed that ROR*α* plays a critical role in the regulation of the survival and differentiation of Purkinje cells from embryonic development throughout adulthood [98, 100–103]. Deletion of ROR*α* in Purkinje cells between postnatal days 10–21 revealed that continued expression of ROR*α* is necessary after neuronal maturation to maintain mature morphological and innervation characteristics in the adult Purkinje cells [103]. Loss of Purkinje cells was also observed in >6 months-old male *RORα*^+/*sg*^ mice and to a much smaller degree in *RORα*^+/*sg*^ females. The marked, age-related Purkinje cell death in *RORα*^+/*sg*^ male mice has been linked to a premature decrease in circulating sex steroids, which have been shown to be neuroprotective, and appeared not to be due to changes in cerebellar neurosteroids [104]. Genome-wide gene expression studies showed that ROR*α* regulates the expression of a number of genes linked to Purkinje cell maturation, particularly dendritic differentiation, and the glutamatergic pathway [98]. ChIP analysis demonstrated that ROR*α* directly controls the transcription of several of these genes, including the glutamate transporter *Slc1a6*, the calmodulin inhibitor *Pcp4*, and the IP3 receptor (*Itpr1*). Several of the ROR*α* target genes were found to be also down-regulated in Ski family transcriptional corepressor 2 (Skor2)-deficient mice; however, ROR*α* expression was not altered in these mice [105]. It was proposed that ROR*α* and Skor2 cooperate in regulating Purkinje cell differentiation and gene expression. In Purkinje cells, ROR*α* also directly regulates the expression of sonic hedgehog (Shh)[98], which is required for the proliferation and survival of cerebellar granule precursor cells through its activation of Gli transcription factors. The degeneration of cerebellar granule cells observed in ROR*α*-deficient mice can at least in part be attributed to this loss in Shh production.

Accumulating evidence indicates a role for ROR*α* in several neuropsychiatric disorders, including autism spectrum and bipolar disorder (ASD), schizophrenia, depression, and posttraumatic stress syndrome [106–117]. Studies demonstrating that the expression of *RORA* was reduced in sections of cerebellum and cortex of autistic subjects and observations showing differential methylation of the *RORA* gene in lymphoblastoid cells from autistic and nonautistic siblings supported a role of ROR*α* in the development of ASD [107]. This, together with reports showing reduced number and size of Purkinje cells (PC) in the majority of cerebellar specimens from ASD patients, as was observed in ROR*α*-deficient mice (Chugani 2014), is consistent with a link between ROR*α*, its regulation of Purkinje cell survival and differentiation, and ASD. A connection between ROR*α* and ASD is further supported by genome-wide ChIP-Seq analysis showing that in human neuronal cells, ROR*α* was associated with the promoter region of a number of ASD-associated genes, including ataxin binding protein (*A2BP1*), neuroligin 1 (*NLGN1*), and aromatase (*CYP19A1*) [118, 119]. Several of the genes down regulated in ROR*α*-deficient neuronal cells, including aromatase, were also found to be repressed in the frontal brain of individuals with ASD. The positive regulation by ROR*α* of aromatase, which converts testosterone to estrogen, is intriguing because estrogen has been reported to enhance ROR*α* expression and to exhibit a neuroprotective effect, as reported for ROR*α*. Thus, RORα, aromatase, and estrogen might be part of a positive regulatory pathway. Therefore, reduced expression of aromatase and estrogen production in ROR*α*-deficiency might excerbate Purkinje cell death and enhance the risk for ASD. Loss of Purkinje cells was also observed in >6 months-old male *RORα*^+/*sg*^ mice and to a much lesser degree in *RORα*^+/*sg*^ females. The marked, age-related Purkinje cell death in *RORα*^+/*sg*^ male mice was linked to a premature decrease in circulating sex steroids, which have been shown to be neuroprotective, and not due to changes in cerebellar neurosteroids [104]. Since both ROR*α* and estrogen exhibit neuroprotective effects, this pathway might help to explain why *RORα*^+/*sg*^ female mice are less susceptible to Purkinje cell loss during aging. Further indications for a link between ROR*α* and ASD came from a recent study demonstrating that the microRNA MIR137, which has been implicated in ASD and schizophrenia, targets the 5’-UTR of ROR*α* [108]. ROR*α* has also been reported to have neuroprotective functions in neurons and astrocytes during hypoxia [120, 121]. Protecting brain cells from the damaging effects of injury and stress might be an important function of ROR*α* and be relevant to several brain disorders.

ROR*β* is highly expressed in the suprachiasmatic nucleus, the retina and pineal gland, and has been implicated in the regulation of circadian, motor, and visual functions [3, 122, 123]. ROR*β* deficient mice displayed motor (“duck gait,” hind paw clasping reflex), olfactory deficits, reduced anxiety and learned helplessness-related behaviors, and alterations in circadian behavior [124]. Examination of ROR*β* expression during embryonic and postnatal development of the mouse neocortex showed that after E16.5 ROR*β* transcripts increasingly localized to the primary sensory areas and reached peak expression at P10 with strongest expression in the primary somatosensory, auditory, and visual areas [125, 126]. This developmental pattern of expression was similar to that reported for rat neocortex [127]. A possible connection was found between ROR*β* expression levels and the control of cytoarchitectural patterning of neocortical neurons during mouse development [128]. GWAS studies revealed an association between a series of *RORB* genetic variants with schizophrenia, and bipolar I disorder [113, 116, 129, 130] and between ROR*β* expression in the temporal cortex and verbal intelligence [131]. Similarly, a syndrome characterized by moderate facial dysmorphy, mental retardation, epilepsy, speech delay, and autistic behaviour in patients with a 9q21 deletion at the *RORB* locus identified ROR*β* as a strong candidate for this neurological disorder [132–134]. Prenatal ethanol exposure has been reported to lead to neurobiological damage in early development. Newborns prenatally exposed to alcohol show neuroanatomical defects in the neocortex and an abnormal neocortical expression pattern of ROR*β* that is associated with mental and intellectual dysfunction, and behavioral and motor deficits [135].

Recent studies showed that the ROR*β*1 and ROR*β*2 isoforms exhibit distinct patterns of expression during retinal development [136, 137]. ROR*β* was shown to play a critical role in regulating retinal progenitor proliferation and differentiation [123, 137–139]. *RORβ1*-deficient mice lack amacrine and horizontal interneurons, cells important for the integration of visual information, suggesting that ROR*β*1 is critical for the differentiation of retinal progenitors into these interneurons [136]. This involves direct transcriptional regulation of Ptf1a, a key factor required for the generation of amacrine and horizontal cells, by ROR*β*1. Re-expression of ROR*β*1 was able to rescue amacrine differentiation in *RORβ* null mice. Retinal progenitors can also differentiate into two morphologically, developmentally, and functionally distinct photoreceptors, rods and cones. Rods function in dim light, while cones mediate daylight, and in most mammals, color vision. Mice lacking ROR*β* lose rods, but overproduce primitive S cones that lack outer segments [139]. ROR*β*1 and ROR*β*2 control rod cell differentiation through its transcriptional regulation of neural retina leucine zipper factor (*NRL*), a transcription factor that promotes the differentiation of retinal progenitors into the rod cell lineage, while suppressing the cone cell lineage [137, 139, 140]. The lack of rod photoreceptors in *RORβ* null mice is in part due to the loss of NRL expression. This can be reversed by re-expression of NRL in these mice [139]. ROR*β*2 is expressed in rod photoreceptors and its transcription was shown to be directly regulated by NRL. Thus, ROR*β*2 and NRL form two positive feedback loops that synergistically promote the commitment to a rod cell lineage [137]. In addition to these roles, *RORβ*-deficient mice fail to induce S opsin appropriately during postnatal cone development, suggesting a function for ROR*β* in morphological maturation of cone photoreceptors [123]. ROR*β* was found to activate the S opsin gene (*Opn1sw*) expression through binding sites in the upstream promoter region.

## 5. Role of RORs in Cancer

Very little is known about the role of ROR*β* and ROR*γ* in cancer. Initial analysis of ROR*γ* knockout mice revealed that these mice develop T-cell lymphomas within the first months after birth that rapidly metastasize to liver and spleen [141]. The mechanism underlying the development of thymic lymphomas in ROR*γ* null mice has yet to be elucidated. A recent study reported that low levels of ROR*γ* mRNA expression in somatotroph adenomas was associated with reduced E-cadherin expression and increased epithelial mesenchymal transition (EMT), and increased tumor size and invasiveness [142]. Similarly, higher expression of ROR*γ* correlated with longer metastasis-free survival in breast cancer [143]. With respect to ROR*β* and cancer, a recent study reported that *RORB* is overexpressed in primary leiomyosarcomas, the most common type of uterine sarcoma [144]. In contrast, *RORB* expression was found to be highly down-regulated in both serous and endometrioid types of endometrial cancer [145].

A number of studies reported that ROR*α* expression is significantly down-regulated during tumor development and progression, while expression of exogenous ROR*α* inhibited cell proliferation and tumor growth [146–151]. Reduced ROR*α* expression has been observed in colorectal and mammary carcinomas and found to be associated with poorer prognosis in hepatocellular and breast carcinoma patients [146, 147, 150, 152, 153]. In addition, silencing ROR*α* in mammary epithelial cells significantly enhanced cell proliferation in ductal epithelial cells and promoted side branching of mammary ducts, suggesting that ROR*α* has an important role in mammary gland branching morphogenesis [150]. Conversely, restoring ROR*α* expression in cultured breast cancer cells was shown to inhibit cell migration and suppress tumor growth and metastasis in nude mice. This was accompanied by enhanced expression of semaphorin 3F (*SEMA3F*), a tumor suppressor that inhibits tumor growth and invasiveness. ROR*α* was shown to regulate *SEMA3F* transcription directly through ROREs in its promoter region [150]. A different study indicated a role for SPARC, which is critical in the regulation of cell growth and adhesion, in the anti-tumor and anti-proliferation effects of ROR*α* in human hepatoma cells [154]. *SPARC* was found to be a direct target gene of ROR*α*. Treatment of colon carcinoma HCT116 cells with DNA-damage agents led to a p53-dependent increase in ROR*α* expression that is directly mediated through functional p53 response elements in the *RORa* promoter [155]. ROR*α* itself stabilized p53 by inhibiting its ubiquitination and enhanced *p53* transcription in a HAUSP/Usp7-dependent manner leading to increased apoptosis. The connection between ROR*α* and p53 was supported by a report demonstrating that treatment of hepatocellular carcinoma cells with an ROR*α* agonist enhanced p53 stability [156]. This increase was shown to involve elevated *SOX4* transcription, a gene critically involved in MDM2-dependent regulation of p53 stability. Another role for ROR*α* in cancer was revealed by a recent study demonstrating that ROR*α* expression was decreased in tumor tissues compared to adjacent normal tissues in human hepatocellular carcinoma patients and that this was associated with a change in glucose metabolism [157]. ROR*α* was shown to inhibit pyruvate dehydrogenase kinase 2 (PDK2) expression and phosphorylation, thereby promoting aerobic glycolysis rather than oxidative phosphorylation, whereas reduced ROR*α* expression in tumor cells promotes oxidative phosphorylation and tumor cell growth.

Several studies revealed a role for a noncanonical ROR*α* pathway in cancer that does not involve RORE binding, but in which ROR*α* functions as a transcriptional cofactor. In colon carcinoma cells, ROR*α* was shown to bind *β*-catenin directly and inhibit *β*-catenin-mediated transcriptional activation of the target genes, cyclin D1 (*CCND1*) and c-myc (*MYC*), resulting in repression of cell proliferation and migration [158]. ROR*α* was found to be recruited to the lymphoid enhancer-binding factor 1 (LEF1)-binding sites of the LEF1-target gene, *CCND1*, together with LEF1 and *β*-catenin. ROR*α* interacted with residues within the armadillo repeat domains of *β*-catenin, which function as binding sites for a subset of coactivators. The interaction of ROR*α* with *β*-catenin required the N-terminus of ROR*α* and was dependent on the phosphorylation of Ser35 by protein kinase C*α* (PKC*α*) activated by the noncanonical Wnt pathway. Interestingly, in many colorectal carcinomas phosphorylation of ROR*α* was reduced. In another noncanonical pathway, ROR*α* interacts with the heptad repeat and marked box region of the transcription factor E2F1 and suppressed E2F1-regulated transcription and cell cycle progression in epithelial cells [159]. In mammary ducts, ROR*α* levels inversely correlated with the expression of E2F1 target genes and cell proliferation. Binding of ROR*α* was shown to inhibit E2F1 acetylation and its DNA-binding activity by enhancing its interaction with histone deacetylase 1 (HDAC1). Knockdown of HDAC1 or inhibition of HDAC activity partially reversed the repression of E2F1 activity by ROR*α*. In contrast to its growth inhibitory effects, ROR*α* was shown to enhance the proliferation in mammary carcinoma MCF7 cells and significantly induced the expression of aromatase mRNA by binding an RORE in the aromatase promoter region [160]. It was proposed that the increase in aromatase expression by ROR*α* accelerates the local production of estrogen, which then enhances the proliferation of breast cancer cells.

## 6. RORs in the Regulation of Metabolism

Both ROR*α* and ROR*γ* have been implicated in the control of energy homeostasis and the regulation of several lipid and glucose metabolic genes [3, 22, 24, 161–169]. Regulation of energy homeostasis is a complex process that involves multiple interrelated glucose and lipid metabolic pathways in many organs and is controlled by the circadian clock, gut microbiota, and by the endocrine, immune and nervous systems [170–173]. This has made it difficult to determine whether the metabolic changes observed in ROR-deficiency are cause or effect. ROR*α*-deficient (*staggerer*) mice were shown to be protected against high fat diet (HFD)-induced metabolic syndrome as indicated by reduced weight gain, adiposity and hepatic steatosis, and improved insulin sensitivity [161, 174, 175]. Adipocytes in ROR*α*-deficient mice fed a high fat diet accumulated considerably less lipid and the infiltration of inflammatory macrophages and expression of several inflammatory genes, including interleukin 6 (*Il6*), Toll-like receptor 8 (*Trl8*), and chemokine (C-C motif) ligand 8 (*Ccl8*), were greatly diminished [174]. Interleukin-1 receptor antagonist (*Il1rn*) was among the genes most dramatically repressed in white adipose tissue (WAT) of ROR*α*-deficient mice. This gene has been implicated in the regulation of obesity and insulin resistance, suggesting that the reduced susceptibility to metabolic syndrome in ROR*α*-deficient mice might at least in part be attributed to *Il1rn* repression [174, 176]. WAT-associated inflammation plays a critical role in the development of metabolic syndrome [172, 177]. The reduced inflammation observed in *RORα*-deficient mice might be in part responsible for the improved insulin sensitivity in these mice. A role for RORα in the regulation of insulin sensitivity is supported by a study showing an association between a single nucleotide polymorphism in *RORα* (rs7164773) and an increased risk for type 2 diabetes in the Mexico Mestizo population [178]. A recent study showed that the expression of several thermogenic genes, such as uncoupling protein 1 (*Ucp1*) and deiodinase 2 (*Dio2*), markers of brown adipose tissue (BAT), was enhanced in adipose tissue from ROR*α*-deficient mice. This was associated with increased expression of the histone-lysine N-methyltransferase 1 (*Ehmt1*), a gene that controls BAT specification and maintenance [175, 179]. The greater cold-tolerance of *RORα*-deficient mice appears to be related to the increased expression of these genes, leading to increased oxygen consumption and heat generation from lipid oxidation that likely contributes to the improved energy homeostasis and insulin-sensitivity observed in these mice. Both ROR*α* and ROR*γ* have been shown to be induced during adipocyte differentiation in 3T3-L1 cells [180]; however, exogenous expression of ROR*α* inhibits adipocyte differentiation in 3T3-L1 cells, as indicated by the reduced induction of fatty acid binding protein 4 (*Fabp4*), perilipin 1 (*Plin1*) and fatty acid synthase (*Fasn*) [181].

In addition to WAT, loss of ROR*α* induces changes in gene expression in macrophages and liver. Disruption of ROR*α* in macrophages leads to diminished expression of cholesterol 25-hydroxylase (Ch25h), which converts cholesterol to 25-hydroxycholesterol, and reduced phagocytosis [182, 183]. Interestingly, addition of 25-hydroxycholesterol was able to reverse the inhibition of phagocytosis in ROR*α*-deficient macrophages suggesting a link between oxysterol metabolism and the regulation of phagocytosis. In the liver, the expression of a large number of genes related to lipid and glucose metabolism were found to be down-regulated in ROR*α*-deficient mice fed a HFD [174]. These included phosphoenolpyruvate carboxykinase (*Pepck*) and glucose-6 phosphatase (*G6pc*), which play a role in gluconeogenesis, fibroblast growth factor 21 (*Fgf21*), which is an important regulator of glucose and lipid homeostasis, and genes involved in triglyceride synthesis and storage, such as glycerol-3-phosphate acyltransferase (*Gpam*), perilipin 2 (*Plin2*), monoacylglycerol O-acyltransferase 1 (*Mogat1*), and cell death-inducing DFFA-like effector a (*Cidea*) [154, 166, 174, 184]. In addition, the hepatic expression of several genes involved in sterol and bile acid metabolism, including cytochrome P450 8b1 (*Cyp8b1*), *Cyp7b1*, and sulfotransferase *Sul1b1* were significantly diminished in ROR*α*-deficient mice [153, 167, 174, 185]. However, the hepatic expression of sulfotransferase *Sult1e1* was found to be dramatically induced in both male and female *RORα*-, but not in *RORγ*-deficient mice, whereas *Sult2a1*, known to sulfonate bile acids, hydroxysteroid dehydroepiandrosterone, and related androgens, was increased in both ROR*α*- and ROR*γ*-deficient mice, but only in female mice [167]. In contrast, in cultured human hepatocytes and hepatoma HepG2 cells, exogenous expression of ROR*α* induced *SULT2A1*, while ROR*α* knockdown with siRNAs decreased its expression [153]. Moreover, overexpression of ROR*α* inhibited LXR and SREBP expression as well as lipid accumulation in these cells [186]. Adenovirus-mediated overexpression of ROR*α* in liver also reduced triglyceride levels in mice fed a high fat diet. The cause of the discrepancy between the observations in ROR*α*-deficient mice and those in HepG2 and liver overexpressing ROR*α* has yet to be understood. ChIP and promoter analysis indicated that many metabolic genes, including *G6pc, Adfp, Cyp7b1*, citrate synthase (*Cs*), *Cyp2c8, Fgf21*, secreted protein, acidic, cysteine-rich (*Sparc*), *Sult1b1*, and *Sult2a1*, were directly regulated by ROR*α* in HepG2 cells [153, 154, 166, 174, 185, 187, 188]. ROR*α* cistrome data [165] revealed that in liver, ROR*α* was recruited to ROREs in several genes important in glucose homeostasis and lipid metabolism, including *G6pc, Fasn, Pepck1, Apoa1, and Elovl3*, indicating that ROR*α* positively regulates the transcription of these metabolic genes by binding ROREs in their regulatory region.

ROR*α*-deficient mice also display metabolic changes in skeletal muscle that are accompanied by alterations in the expression of several genes [169]. Glucose uptake in skeletal muscle of ROR*α*-deficient mice was enhanced and found to be associated with increased phosphatidylinositol 3-kinase signaling and *Glut4* expression [161, 169]. Expression of a dominant-negative ROR*α* in skeletal muscle C2C12 cells and in skeletal muscle in mice was reported to down-regulate the expression of carnitine palmitoyltransferase-1 (*Cpt1*), caveolin 3 (*Cav3*), and *Abca1* encoding proteins involved in *β*-oxidation and cholesterol homeostasis, and of *Srebp1c* and its downstream targets, *Fas* and *Scd1/2l*, which are involved in lipogenesis [163, 189]. Promoter analysis indicated that *Cav3* and *Cpt1* were directly regulated by ROR*α*. Expression of a dominant-negative ROR*α* in skeletal muscle induced mild hyperglycemia and glucose intolerance and attenuated insulin-mediated phosphorylation of Akt2. The latter contrasts with the increase in Akt2 expression and phosphorylation observed in *RORα*-deficient *sg/sg* mice.

ROR*γ* also plays a role in the regulation of glucose metabolism and insulin sensitivity [164, 165, 168, 190, 191]. *RORγ*-deficient mice were significantly more insulin sensitive and glucose tolerant than WT mice. The euglycemic clamp test revealed that hepatic glucose production was considerably reduced in *RORγ*-deficient mice, whereas ectopic expression of ROR*γ* in *RORγ*-deficient liver tissue or primary hepatocytes increased glucose production [165]. Moreover, the conversion of exogenously administered pyruvate to glucose was significantly lower in *RORγ*^−/−^. The reduced hepatic gluconeogenesis in *RORγ*-deficient mice may be at least partly responsible for the improved insulin sensitivity and glucose tolerance observed in these mice [165, 190]. Loss of ROR*γ* significantly decreased peak expression of several glucose (e.g., *G6pase, Pklr, Glut2, PPARδ*) and lipid (e.g., *Insig2a, Elovl3, Cyp8b1, Sult1e1*) metabolic genes [165, 167, 168, 192]. Conversely, exogenous expression of ROR*γ* in *RORγ*^−/−^ liver tissue by adenovirus significantly increased the expression of *G6pase, Pepck, Gck, Gckr, Ppar*δ*, Pcx*, and *Klf15*. [165]. Together, these observations indicated that ROR*γ* is an important modulator of hepatic gluconeogenesis and glycolysis. ChIP-Seq analysis not only uncovered the consensus sequence of the *in vivo* RORE, but also revealed that ROR*γ* is recruited to the regulatory region of a number of metabolic genes involved in glycolysis and gluconeogenesis, including *G6pase, Pepck, Pklr, Pparδ, Gck, Gckr, Glut2, Gys2, Dlat, Pcx*, and *Klf15* [165]. These data indicated that ROR*γ* positively regulates the transcription of these metabolic genes by binding ROREs in their regulatory region. Promoter analysis further supported that the expression of several of these genes was directly regulated by ROR*γ*. The observations further suggested that the decreased expression of these genes is at least in part responsible for the reduced gluconeogenesis and lower glycogen accumulation and consequently for the improved insulin sensitivity and glucose tolerance observed in *RORγ* null mice. A role for ROR*γ* in the regulation of insulin resistance is supported by studies showing that the level of *RORγ* expression positively correlates with adiposity and insulin resistance in human obese patients [190, 191]. These observations suggest that ROR*γ* antagonists might be beneficial in controlling glucose homeostasis and in the management of metabolic diseases.

In addition to gluconeogenesis, ROR*γ* regulates hepatic lipid metabolism. Loss of ROR*γ* reduced the expression of a number of lipid metabolic genes, including the insulin-induced gene 2a (*Insig2a*), elongation of very long chain fatty acids-like (*Elovl3), Sult2a1, Cyp7b1*, and *Cyp8b1* [153, 167, 168, 185]. ChIP and promoter analysis showed that several of these genes are directly regulated by ROR*γ*. The changes in the expression of these genes were associated reduced levels of triglycerides, cholesterol, and bile acids in liver and blood in ROR*γ*-deficient mice fed a HFD. Lipid and glucose metabolic genes are under a complex control and involve regulation by other transcription factors, including several nuclear receptors, such as Rev-Erb, PPAR, LXR, and CAR. Since some of these receptors interact with similar binding sites, the transcriptional control of several of lipid and glucose metabolic genes likely involves interplay between different nuclear receptor signaling pathways. The best known example of this is the competition of Rev-Erbs with RORs for the same binding sites. Comparison of the ROR*α* and ROR*γ* cistromes from liver indicated that although many genes were selectively regulated by either ROR*α* or ROR*γ*, several genes, including *G6pc, Apoa2, Elovl5*, and *Cry1*, were regulated by both ROR*α* and ROR*γ*, indicating some redundancy between the two RORs in regulating these genes [165].

## 7. RORs and Circadian Rhythm

**Figure 3:**
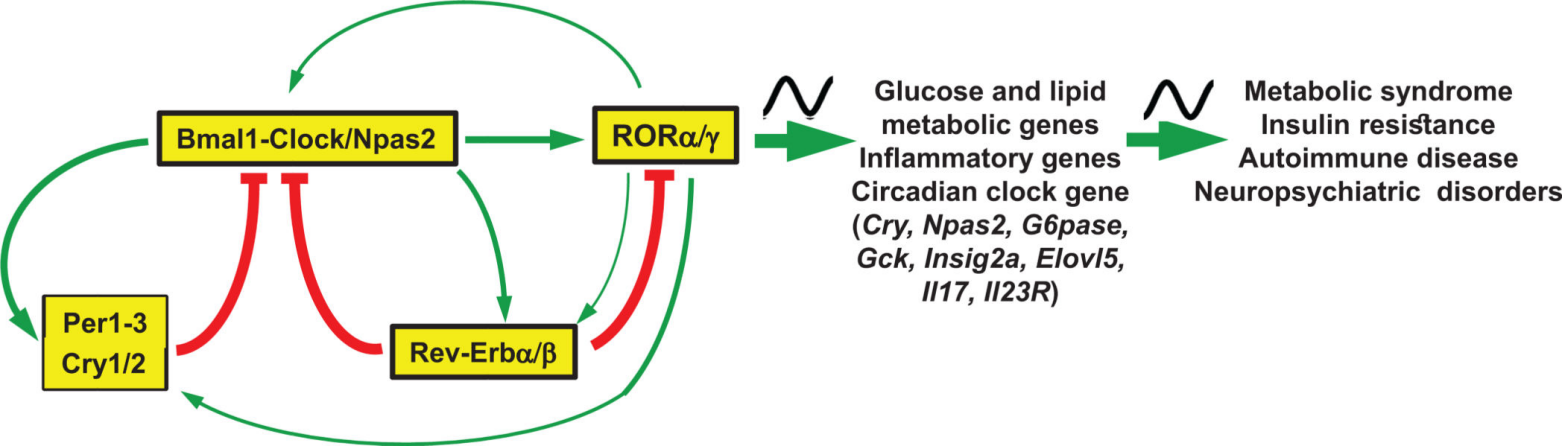
ROR*α* and ROR*γ* function as intermediaries between the circadian clock and its regulation of glucose/lipid metabolic and inflammatory gene expression RORs are linked to the circadian clock at different levels: a) ROR expression is regulated by the circadian clock machinery, including Bmal1, Clock, Rev-Erbs and Cry1; b) RORs are involved in the modulation of clock gene expression, including *Npas2, Clock* and *Rev-Erb*, and participate in the regulation of the rhythmic expression of glucose and lipid metabolic genes as well as inflammatory genes; c) Deficiency in ROR*α* or ROR*β* causes changes in the circadian behavior, which might be linked to neuropsychiatric disorders, while deficiency in ROR*γ* leads to increased insulin sensitivity and glucose tolerance and a lower risk of developing diabetes.

It has been well established that the regulation of the circadian rhythm is interconnected with the diurnal control of behavior, metabolic activities, immune responses, and many other physiological functions. For example, the circadian clock has been shown to regulate the diurnal expression of many lipid and glucose metabolic genes as well as immune response genes [170, 193–195]. It therefore not surprising that disruption of the circadian rhythm has been linked to increased risk for metabolic diseases, including obesity, diabetes, and liver steatosis, as well as several inflammatory and neuropsychiatric disorders [170, 171, 196–201]. In mammals, the suprachiasmatic nucleus (SCN) serves as the central circadian pacemaker that integrates light-dark cycle input and synchronizes the autonomous oscillators in peripheral tissues [170, 171, 196, 197]. The molecular clock machinery consists of several transcription/translation feedback loops in which the heterodimeric complex consisting of brain and muscle ARNT-like (Bmal1) and circadian locomotor output cycles kaput (Clock) or its paralog neuronal PAS domain protein 2 (Npas2) form the positive regulatory loop of the oscillator, whereas two cryptochrome (Cry) and three period proteins (Per) are part of the negative control mechanism. The nuclear receptors Rev-Erb*α* and *β* (NR1D1/2) further regulate the core loop by repressing the transcription of several clock genes, including *Bmal1, Clock* and *Npas2* (Figure 3).

RORs are associated with the circadian clock at several different levels (Figure 3). First, RORs exhibit a rhythmic pattern of expression in several tissues. In particular, ROR*γ* exhibits a robust oscillatory pattern of expression in liver, brown adipose tissue (BAT), pancreatic *β* cells, kidney, and small intestines (jejunum), with peak expression around Zeitgeber Time (ZT) 16–18, whereas ROR*α* exhibits no to moderate oscillation in the SCN and several other tissues [94, 192, 202–205]. ROR*β*2 displays a rhythmic expression pattern in mouse SCN, pineal gland and retina, with a maximum at ZT18 [122, 203, 206, 207]. Several studies showed that *RORa* and *RORc* are regulated by Bmal1/Clock and RevErb. This is supported by data indicating that Bmal1, Clock and Rev-Erb*α/β* were recruited to the E-box and RORE, respectively, in the proximal *RORc* promoter in mouse liver [192, 208, 209]. Moreover, Bmal1 and Clock were able to induce activation of the *RORc* promoter in reporter assays [192, 210]. The *RORc* gene contains two E-box binding sites for Bmal/Clock [192, 204, 208, 211]. Mutation of either E1 or E2 significantly reduced the activation, while the double mutation totally abolished this induction by Clock/Bmal1. The activation of the *RORc* promoter by Clock/Bmal1 was repressed by Cry1 and correlated with changes in chromatin accessibility at the *RORc* promoter. Rev-Erbs, rather than Bmal1, regulate the rhythmic expression of *RORc* [210]. This is supported by data showing that in *Bmal1* KO mice, the hepatic expression of ROR*γ* is greatly enhanced particularly at ZT4–8, thereby largely abolishing the robust rhythmic expression pattern of ROR*γ* [210]. The increase in ROR*γ* mRNA expression appeared largely due to the loss of RevErb expression in *Bmal1* KO liver, which subsequently abolished the repression of *RORc* by Rev-Erb at ZT4–8.

A second association between RORs and the circadian clock is their participation in the diurnal regulation of a number of clock genes, including *Bmal1, Npas2, Clock, Rev-Erbα*, and *Cry1* [1, 3, 24, 171, 192, 202, 211, 221]. Exogenous expression of ROR*γ*, as well as of ROR*α*, in Hepa1–6 cells enhanced the endogenous expression of *Cry1, Bmal1, E4bp4, Clock, Npas2*, and *Rev-Erbα*, whereas treatment with an ROR*γ* antagonist inhibited their induction [24, 192, 214]. ROREs have been identified in these clock genes [24, 192, 211, 214, 218]. Reporter gene and mutation analysis indicated that RORs are involved in the transcriptional regulation of these genes [24, 192, 214–217]. Rev-Erbs, which can compete with RORs for RORE binding, inhibited this activation. ChIP-Seq and ChIP-QPCR analyses further supported the association of RORs with these ROREs *in vivo*, consistent with the conclusion that these clock genes are directly regulated by RORs. The transcriptional mediator, RIP140, has been shown to be recruited by ROR*α* to the *Bmal1* promoter, suggesting that it is involved in mediating the transactivation of *Bmal1* by ROR*α* [222].

One might predict that the rhythmic expression of RORs leads to a rhythmic expression of ROR target genes. Indeed, several studies demonstrated that, in addition to clock genes, ROR*γ* also participates in the diurnal regulation of several metabolic genes. Loss of ROR*γ* significantly decreased peak expression of several glucose (e.g., *G6pase, Pklr, Glut2, PPARδ*) and lipid (e.g., *Insig2a, Elovl3, Cyp8b1, Sult1e1*) metabolic genes [165, 167, 168, 192]. ChIP analysis showed a ZT-dependent association of ROR*γ* with ROREs in several of these genes. The transcriptional mediator, Prospero-related homeobox 1 (Prox1), which functions as a co-repressor of RORs as well as several other nuclear receptors, was shown to participate in the diurnal regulation of hepatic lipid/glucose metabolism by RORs [223, 224]. ROR*γ*-deficient mice exhibited a significantly greater insulin sensitivity and glucose tolerance than WT mice particularly at ZT4–6. Moreover, the conversion of exogenously administered pyruvate to glucose was significantly lower in *RORc*^−/−^ mice particularly at ZT4–6. Together these findings suggested that ROR*γ* participates in the diurnal regulation of hepatic lipid metabolism, gluconeogenesis and insulin sensitivity. These studies further suggest that ROR*γ* functions as an intermediary between the circadian clock machinery and its regulation of glucose and lipid metabolism.

Recent observations uncovered a connection between RORs and the circadian control of immune functions. ROR*γ*t was found to play a role in the diurnal regulation of Th17 differentiation by the circadian clock [225]. In Th17 cells, ROR*γ*t is expressed at significantly higher levels at daytime than at nighttime. This diurnal pattern of expression was found to be related to an increase in the daytime expression of Rev-Erb by Bmal1/Clock, which results in repression of *NFIL3* transcription. Since NFIL3 functions as a repressor of ROR*γ*t transcription, its repression during daytime alleviates its inhibition of *RORγt* transcription leading to enhanced ROR*γ*t expression. Another study demonstrated that in the ileum, ROR*α* regulates the diurnal expression of several genes associated with TLR signaling [94]. Analysis of gene expression profiles of mucosal biopsies from healthy individuals and patients with inflammatory bowel diseases (IBD) showed that the expression of several circadian genes, including *ARNTL2*. *NPAS2, PER1*, and *RORA*, was upregulated in IBD patients, consistent with a role for these proteins in this pathophysiology [226]. Together, these studies indicate that ROR*α* and ROR*γ* function as a link between the circadian clock and its regulation of various inflammatory pathways and provide a possible mechanism by which disruption of the circadian rhythm is associated with an increased risk of inflammatory diseases.

Clinical studies have indicated an important association between abnormalities in circadian rhythms and patients with mood and neuropsychiatric disorders. Alterations in circadian behavior observed in mice deficient in either ROR*α* or ROR*β* receptor [122, 124, 216] and associations between SNPs in *RORA* and *RORB* with an increased risk for several neuropsychiatric disorders, including autism spectrum (ASD) and bipolar disorder, schizophrenia, depression, and posttraumatic stress syndrome [106–117, 129, 130], would be consistent with a link between disturbance in the circadian rhythm and these pathologies.

## 8. ROR (ant)agonists

**Figure 4:**
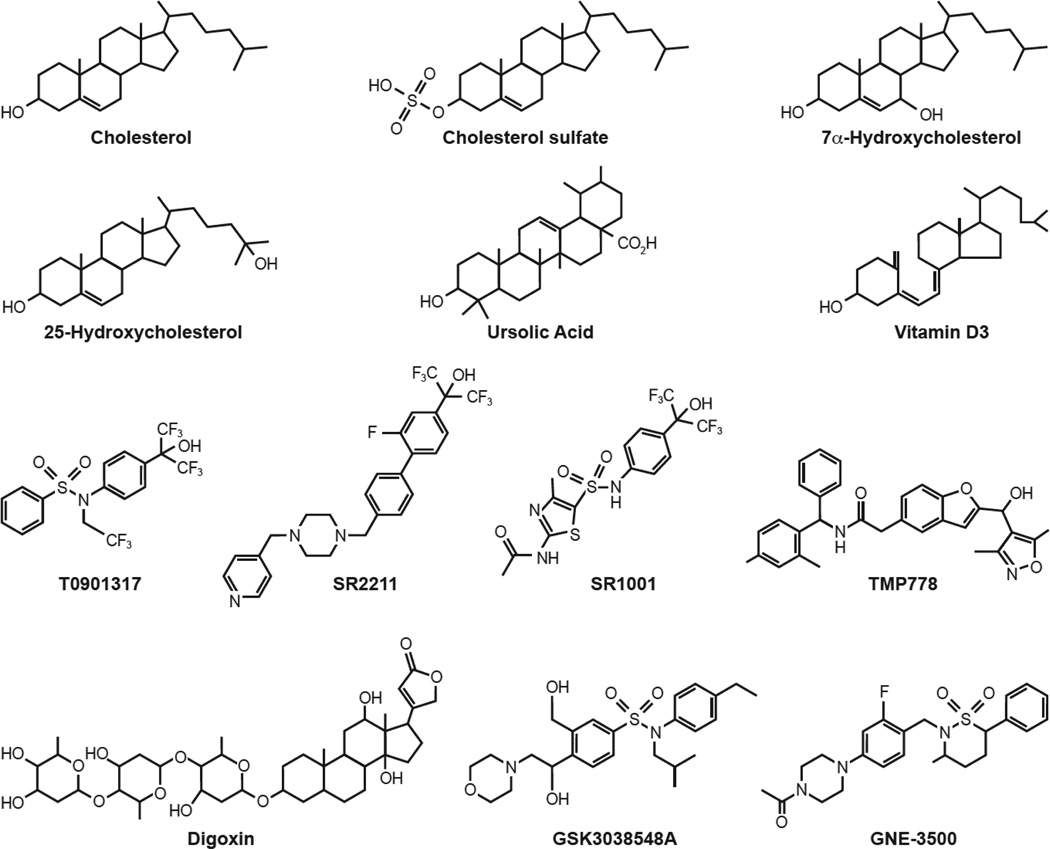
Chemical structure of several ROR*α/γ* inverse agonists and (ant)agonists T0901317, SR1001, and 7*α*-hydroxycholesterol function as inverse agonists of both ROR*α* and ROR*γ*; cholesterol, cholesterol sulfate, and 25-hydroxycholesterol act as ROR*α* and/or ROR*γ* agonists; all other compounds have been reported to function as an inverse agonist or antagonist of ROR*γ*.

There has long been debate about whether RORs function as constitutively active receptors or whether their activity is regulated by (endogenous) ligands that function as an agonist or active antagonist (referred to as inverse agonist) or neutral antagonist [227]. Kallen, Stehlin-Gaon, and co-workers provided the first evidence for the hypothesis that RORs function as ligand-dependent transcription factors [33, 228, 229]. Crystal structure analysis revealed that cholesterol and cholesterol sulfate (Figure 4) bind the ligand-binding pocket of ROR*α* and act as ROR*α* agonists [33, 228]. Similarly, several retinoids were found to interact with the ligand-binding pocket of ROR*β* and to function as inverse agonists of ROR*β* as well as ROR*γ* [229]. Subsequent studies identified a series of oxysterols as ligands for ROR*α* and ROR*γ* [25, 32, 230–233]. For example, 7*α*-hydroxycholesterol and 24(R)-hydroxycholesterol (Figure 4) were shown to function as inverse agonists, while 25-hydroxycholesterol, 20(*α*)-hydroxycholesterol, 22(R)-hydroxycholesterol, and 7*α* and 7*β* 27-hydroxycholesterol act as agonists in mammalian cells. A search for additional ROR ligands led to the discovery of a number of other small molecule modulators of ROR*γ* [25, 32, 234–242]. The synthetic LXR agonist T0901317 was found to interact with both ROR*α* and ROR*γ* and to act as an inverse agonist [234]. Through chemical modification of T0901317, Burris and Griffin and coworkers identified a series of related ROR agonists and inverse agonists, such as SR2211 and SR1001 (Figure 4), which do not bind LXR [25, 237, 243]. Some of these compounds interacted with both ROR*α* and ROR*γ*, while others were ROR*α*- or ROR*γ*-selective [25, 234, 237]. Ursolic acid, a pentacyclic triterpene acid found in many plants, and several vitamin D metabolites, including 20-hydroxyvitamin D, were shown to exhibit ROR*γ* antagonist activity [35, 244, 245]. Evidence was provided suggesting that these vitamin D metabolites were able to bind the ROR*γ* LBD. A high throughput screen for ROR*γ* ligands led to the identification of the cardiac glycoside, digoxin (Figure 4), and several of its analogs as ROR*γ* antagonists [246]. Subsequently, other investigators set out to discover additional ROR*γ* ligands [32]. This led to the identification of several series of high affinity ROR*γ* inverse agonists, including various sulfonamides, such as GSK3038548A and GNE-3500 (Figure 4)[43, 240–242, 247, 248]. For a comprehensive review of small molecule ligands that interact with and modulate ROR receptors, we refer to several recent reviews [2, 25, 32, 249].

**Figure 5:**
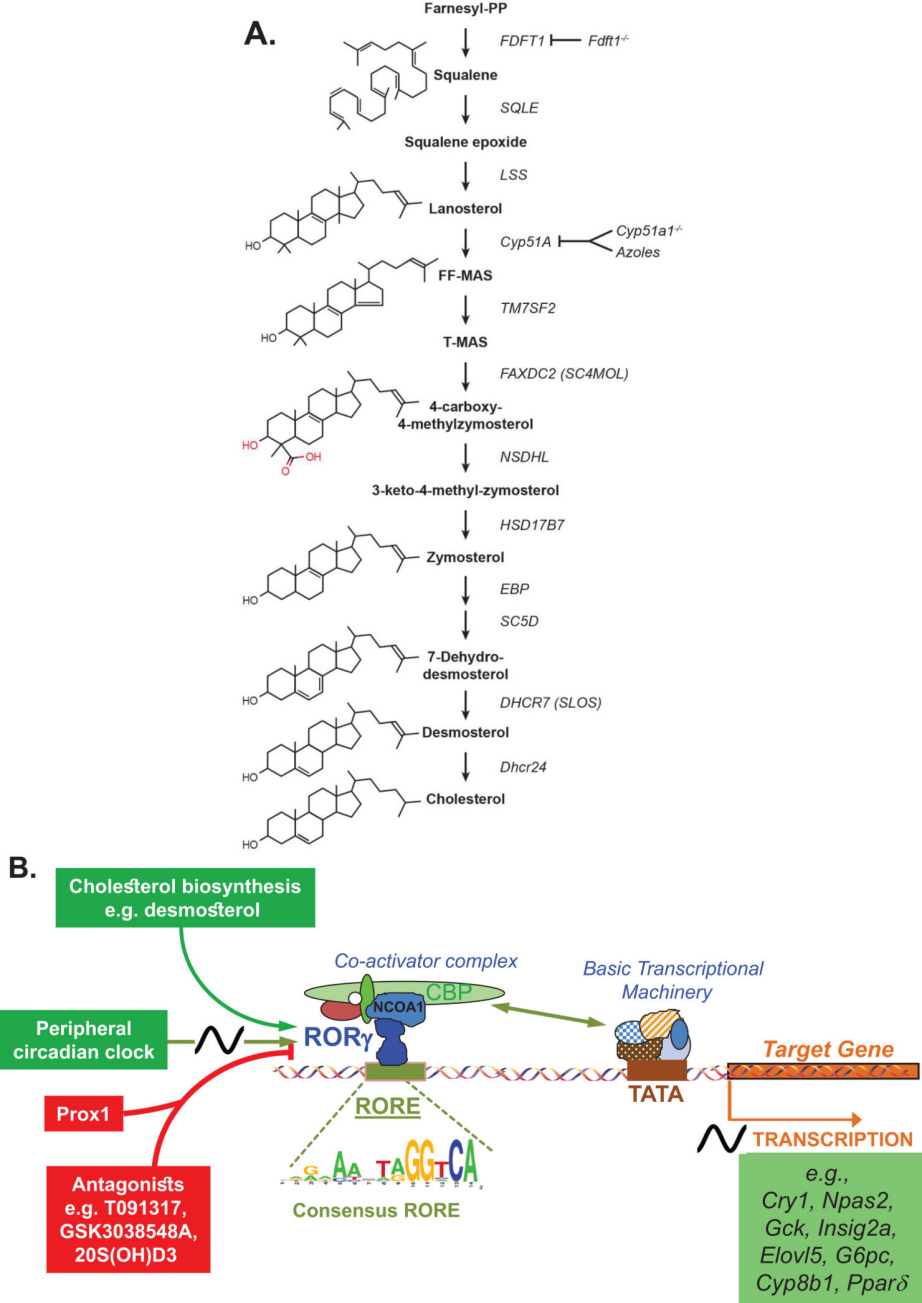
Metabolites of the cholesterol biosynthetic pathway function as endogenous ROR*γ* agonists A. Shown is a schematic view of the cholesterol synthetic pathway. Zymosterol and desmosterol are among the ROR*γ* agonists with the highest affinity. Deficiency in Fdft1 or Cyp51A1, enzymes acting upstream in the cholesterol biosynthetic pathway, inhibit the synthesis of downstream ROR*γ* agonists subsequently leading to reduced ROR*γ*t activation and Th17 differentiation. FDT1, Farnesyl-Diphosphate Farnesyltransferase 1; SQLE, Squalene Epoxidase; LSS, Lanosterol Synthase; TM7SF2, Transmembrane 7 Superfamily Member 2 (C-14 Sterol Reductase); FAXDC2/SC4MOL, Fatty Acid Hydroxylase Domain Containing 2/Methylsterol Monooxygenase 1; NSDHL, NAD(P) Dependent Steroid Dehydrogenase-Like; HSD17B7, Hydroxysteroid (17-Beta) Dehydrogenase 7; EBP, Emopamil Binding Protein (Sterol Isomerase); SC5D, Sterol-C5-Desaturase; DHCR7, 7-Dehydrocholesterol Reductase; DHCR24, 24-Dehydrocholesterol Reductase. B. Schematic view of ROR*γ*-mediated transcriptional activation of target genes by endogenous sterol agonists and its inhibition by antagonists. The circadian clock regulates ROR*γ* expression and as a consequence the expression of ROR*γ* target genes. Prox1 modulates ROR*γ* transcriptional activity. The in vivo consensus RORE derived from ChIP-Seq analysis using liver tissue and an anti-ROR*γ* antibody, is shown.

Recently, the connection between sterols and their modulation of ROR activity was further strengthened by studies showing a link between the cholesterol biosynthetic pathway (Figure 5A) and the regulation of ROR*γ* activity [34, 35]. These studies demonstrated that several intermediates of the cholesterol biosynthetic pathway were able to function as endogenous agonists of ROR*γ*t. Zymosterol and desmosterol were among the most effective sterols activating ROR*γ*, exhibiting EC50s of 0.11 and 0.08 *µ*M, respectively, while cholesterol exhibited a much lower affinity for ROR*γ*. These sterols enhanced ROR*γ* transcriptional activity as well as the recruitment of coactivators. In addition, these sterols were able to enhance Th17 differentiation and increase the expression of IL-17A [34, 35]. Characterization of lipid-bound ROR*γ* complexes immunoprecipitated from mammalian cells supported the concept that cholesterol biosynthetic intermediates function as endogenous ROR*γ* ligands [35]. The connection between ROR*γ* and sterol metabolism was further supported by studies showing that changes in the expression of enzymes involved in the cholesterol biosynthetic pathway were able to modulate ROR*γ* activity. For example, ROR*γ* transcriptional activity was lost in Fdft1-deficient cells lacking squalene synthase, an enzyme acting upstream in the cholesterol biosynthetic pathway [35]. Treatment with azole-type fungicides, such as ketoconazole and clotrimazole, which inhibit the sterol 14*α*-demethylase cytochrome P450, Cyp51a1, an enzyme upstream in the cholesterol biosynthetic pathway (Figure 5A), caused a dramatic reduction in zymosterol and desmosterol levels and a decrease in ROR*γ*-mediated transactivation, Th17 differentiation, and IL-17 expression [34, 250]. Moreover, ROR*γ*-mediated transactivation is greatly diminished in mammalian cells made deficient in Cyp51a1 by shRNA knockdown or germline deletion. Interestingly, several physiological processes that were impaired in ROR*γ*-deficient mice were also affected in Cyp51a1^−/−^ mice [35]; branchial lymph node anlagen were absent in 75% of Cyp51a1^−/−^ mice and the number of IL17RA^+^ and CD4^+^ Lti cells was reduced. In a separate study, mice deficient in the mitochondrial sterol 27-hydroxylase (Cyp27A1), a key enzyme in bile acid synthesis and the production of 27-hydroxy cholesterol, exhibit a reduction in CD4^+^ and *γδ*^+^ T cells and a reduced capacity for Th17 differentiation [233]. These similarities in phenotypic changes are consistent with a link between the cholesterol biosynthetic pathway and ROR*γ* activation. The role of cholesterol synthesis and ROR*γ* activity in Th17 cells was further supported by observations showing that Th17 differentiation is associated with increased cholesterol uptake and biosynthesis and an accumulation of desmosterol that subsequently enhances ROR*γ*t activation and Th17 differentiation. In addition, activation of the TCR pathway, which results in activation of SREBP in favor of sterol-sulfate and cholesterol synthesis, might synergize with ROR*γ* in promoting Th17 differentiation and IL-17 synthesis [34, 251]. Together, these studies suggest that changes in the cholesterol biosynthetic pathway and the level of cholesterol intermediates by diet or cholesterol-lowering drugs might control ROR*γ* activation and as a consequence influence physiological processes regulated by ROR*γ*, including Th17 differentiation. For example, an increase in Th17 cells and IL-17A under hypercholesterolemic conditions might at least in part be due to an increase in endogenous sterol levels and their subsequent activation of ROR*γ*t, while low cholesterol diet might do the inverse. This hypothesis is supported by a study reporting that patients with chronic hepatitis C, which is associated with increased levels of Th17 cells, when placed on a normocaloric, low cholesterol diet showed a significant reduction in Th17 cells and IL-17 levels [252]. Furthermore, treatment with statins, inhibitors of cholesterol synthesis, lead to a reduction in Th17 differentiation and IL-17 production [253].

Several of the sulfated conjugates, such as desmosterol sulfate, have also been shown to activate ROR*γ* at levels twofold higher than the unsulfated sterols. In this context, it is interesting to note that Th17 cell differentiation is accompanied with an increase in the expression of the sulfotransferase, Sult2B1, and reduced expression of the sulfotransferase, STS [34]. This would be consistent with increased synthesis of desmosterol sulfate and ROR*γ* activation and stimulation of Th17 differentiation. Oxysterols exhibit a much lower affinity for ROR*γ* and appear to play a lesser role in modulating ROR*γ* activity in Th17 cells; however, this may depend on the cell type and the type of oxysterol. Interestingly, Cyp7b1 and 3*β*-hydroxysteroid dehydrogenases (3*β*HSDs), which are involved in the hydroxylation or dehydrogenation of sterols, have been reported to be regulated by ROR*α* and ROR*γ* [167, 185] and therefore might affect the formation of certain (oxy)sterols and as a consequence the activation of ROR*γ* [233].

Many of the ROR (ant)agonists have been shown to bind the ligand-binding pocket within the LBD of RORs [2, 32, 33, 228–232]. As has been reported for other nuclear receptors, agonist binding induces a conformational change in the ROR LBD and realignment of helix 12 that allows release of co-repressor complexes and promotes recruitment of co-activator complexes, which then mediate the transcriptional activation by RORs [1]. Particularly, the PLYKELF sequence within the C-terminal activation function (AF) plays a critical role in ROR transactivation activity and mutations in or deletion of this motif result in a dominant-negative ROR [254, 255]. Conversely, binding of ROR antagonists and inverse agonists, such as 25-hydroxycholesterol, inhibits the interaction with co-activators and promote interaction with co-repressors (Figure 5B). A number of co-repressors and coactivators have been identified that mediate ROR-dependent transcriptional activation, including NCOR, SRC1/2, and RIP140 [36, 222, 256].

ROR inverse agonists can inhibit ROR-induced transcriptional activation through different mechanisms. Certain inverse agonists, such as TMP920, have been reported to inhibit ROR*γ* binding to ROREs, whereas the ability of ROR*γ* to bind DNA target sites was mostly preserved with other inverse agonists, such as TMP778 and GSK805 [43]. Interestingly, ROR*γ* cistrome analysis revealed that the latter compounds stabilized ROR*γ*t binding to a number of new genomic sites [43]. The distinct effects by various ligands are likely related to the induction of different conformational changes in ROR*γ* that influence its affinity for different ROREs as well as its interaction with other transcriptional mediators. In addition to Th17 related genes, such as *Il17* [35, 43, 244, 246], ROR*γ* inverse agonists have been reported to inhibit the expression of a number of ROR*γ* target genes, including the clock genes, *Bmal1, Cry1*, and *Npas2*, and several glucose and lipid metabolic genes, such as *G6pase, Insig2a, Elovl3, Gck*, and *PPARδ* [24, 165, 168, 192]. ROR*α* inverse agonists and agonists were shown to, respectively, suppress or induce the expression of the ROR*α* target genes, *G6pase, Fgf21, CS*, and *Npas2* [166, 187, 231].

## 9. Summary

The clear evidence that ROR*γ* activity is regulated by endogenous ligands suggests that this is likely the case also for ROR*α* and ROR*β*. The regulation of ROR*γ* activity by intermediates of the cholesterol biosynthetic pathway suggests that ROR*γ* and the physiological processes controlled by ROR*γ* can be influenced by environmental factors that affect this pathway, including cholesterol-rich or -low diets, environmental agents, such as the azole-type fungicides, and drugs that control cholesterol levels, such as lovastatin. Most importantly, by inhibiting ROR*γ* transcriptional activity and thereby reducing Th17 generation and IL-17A/F production, ROR*γ* inverse agonists may provide a novel strategy in the treatment of various pathologies in which ROR*γ* is implicated, including inflammatory, metabolic, endocrine, and autoimmune diseases [1, 2, 13, 25, 257, 258]. Similarly, ROR*α* antagonists might affect pathologies by inhibiting the generation of ILC2 cells and other physiological functions and be useful in the management of inflammatory, metabolic, and neuropsychiatric disorders [1, 2, 13, 25, 108, 113– 117, 174]. This concept is supported by reports showing that by inhibiting Th17 differentiation and IL-17 production, ROR*γ* inverse agonists suppress Th17 responses in mice and ameliorate the development of experimental autoimmune encephalomyelitis and imiquimod-induced cutaneous inflammation [43, 244, 246, 259]. The beneficial effects of ROR*γ* antagonists may not only be mediated through the inhibition of IL-17A and IL-17F synthesis in Th17 cells, but also by repressing the synthesis of these and other cytokines in ROR*γ*t^+^ innate lymphoid cells (ILC3), and ROR*γ*t^+^ *γδ* T cells, which also play a critical role in several autoimmune and inflammatory diseases [34, 77, 260, 261]. Attenuating ROR*α/γ* activity by antagonist treatment might also be beneficial for the management of metabolic diseases, including metabolic syndrome and insulin resistance [161, 165, 174, 178, 190, 191]. Recently, the ROR*α/γ* inverse agonist SR1001 was shown to suppress insulitis and prevent hyperglycemia in a mouse model of type 1 diabetes [262]. Together, these studies reinforce the potential of ROR antagonists in the management of autoimmune disease, neuropsychiatric and metabolic disorders, and other pathologies.
